# Abdominal and pelvic complications of molecular targeted therapy

**DOI:** 10.1186/1470-7330-14-S1-O37

**Published:** 2014-10-09

**Authors:** Richard M Gore, Robert I Silvers

**Affiliations:** 1 Department of Radiology, North Shore University Health System, University of Chicago, Pritzker School of Medicine, Evanston, IL, USA

## 

Traditional chemotherapy is cytotoxic in nature and acts primarily by eliminating neoplastic cells. Change in tumor size, which is an indicator of change in the number of neoplastic cells, evolved into the radiologic biomarker of treatment response. The infectious, inflammatory, hemorrhagic and neoplastic complications of these therapies have been well described. Significant advances in molecular cytogenetics has led to the development of molecular targeted therapy which selectively acts on tumor cells and modifies their biologic characteristics, by affecting various cellular targets: growth factor receptors, signaling molecules, cell-cycle proteins, molecules that direct apoptosis and angiogenesis. This has required new means of assessing tumor response to therapy. Additionally, a variety of expected and unusual complications can develop in the abdomen and pelvis in these patients. This presentation highlights the imaging features of these complications which may be confusing both radiologically and clinically.

